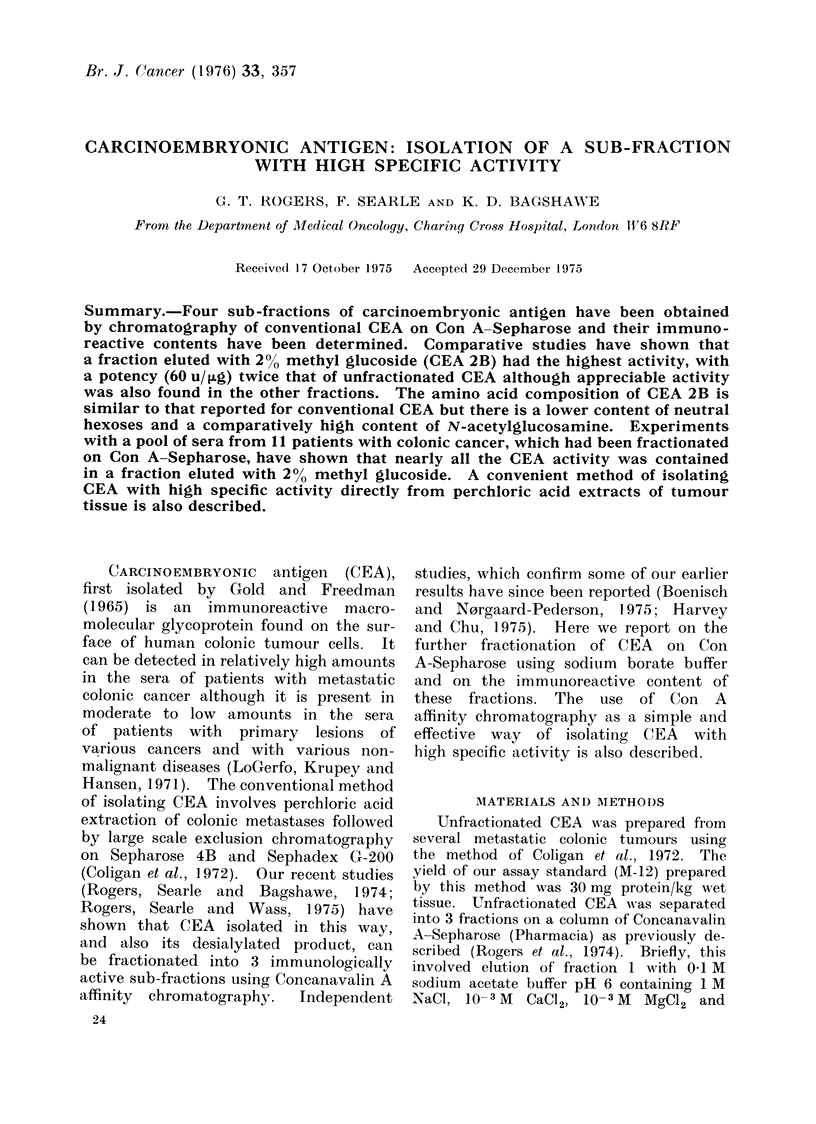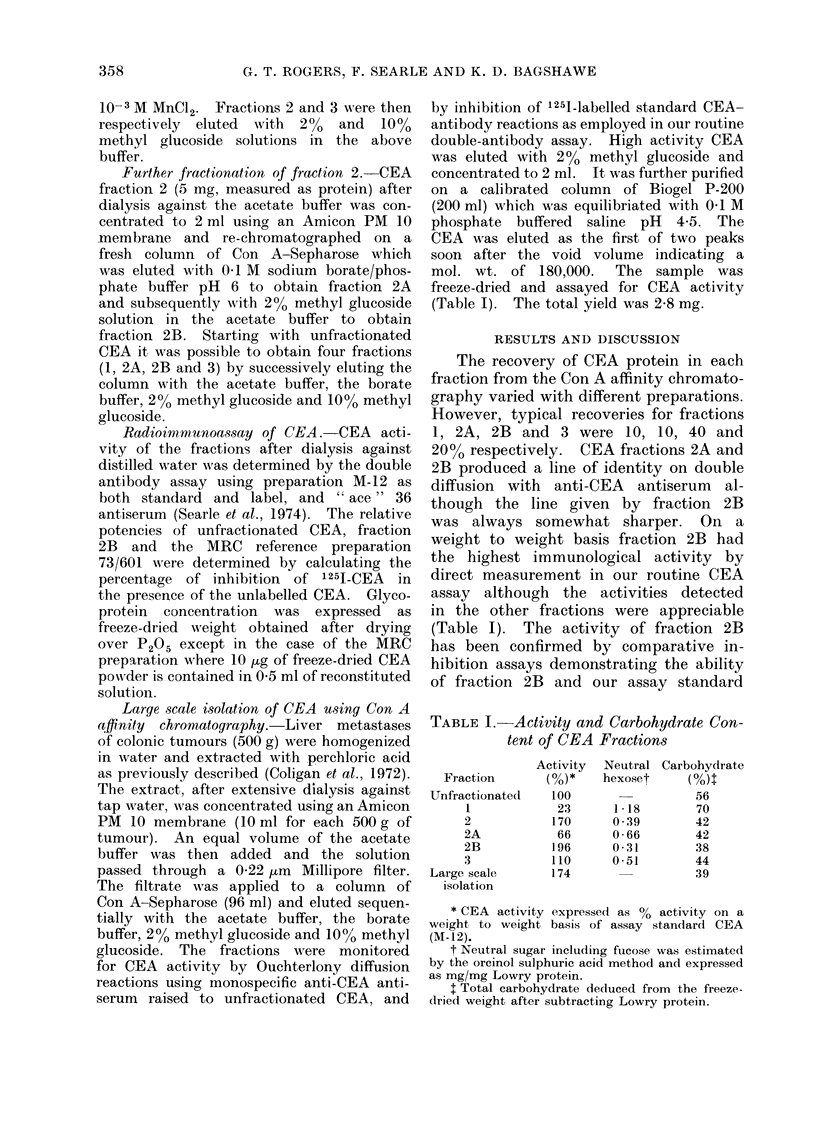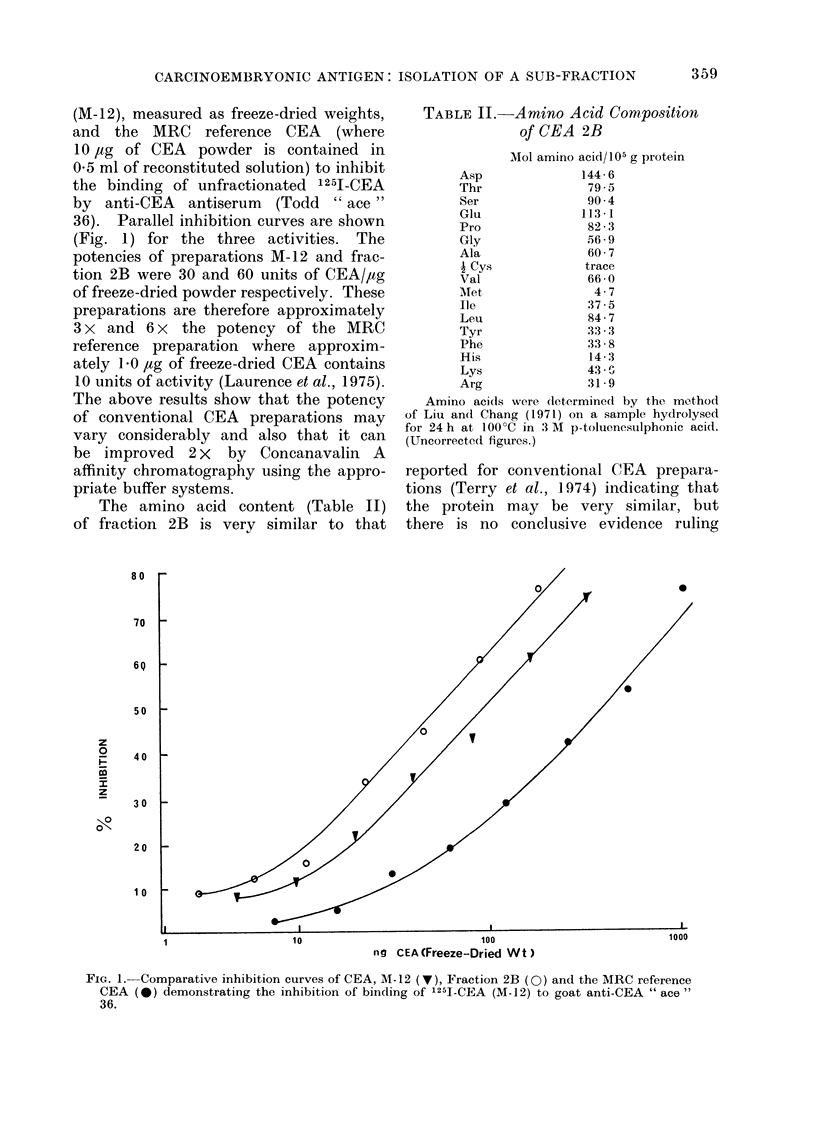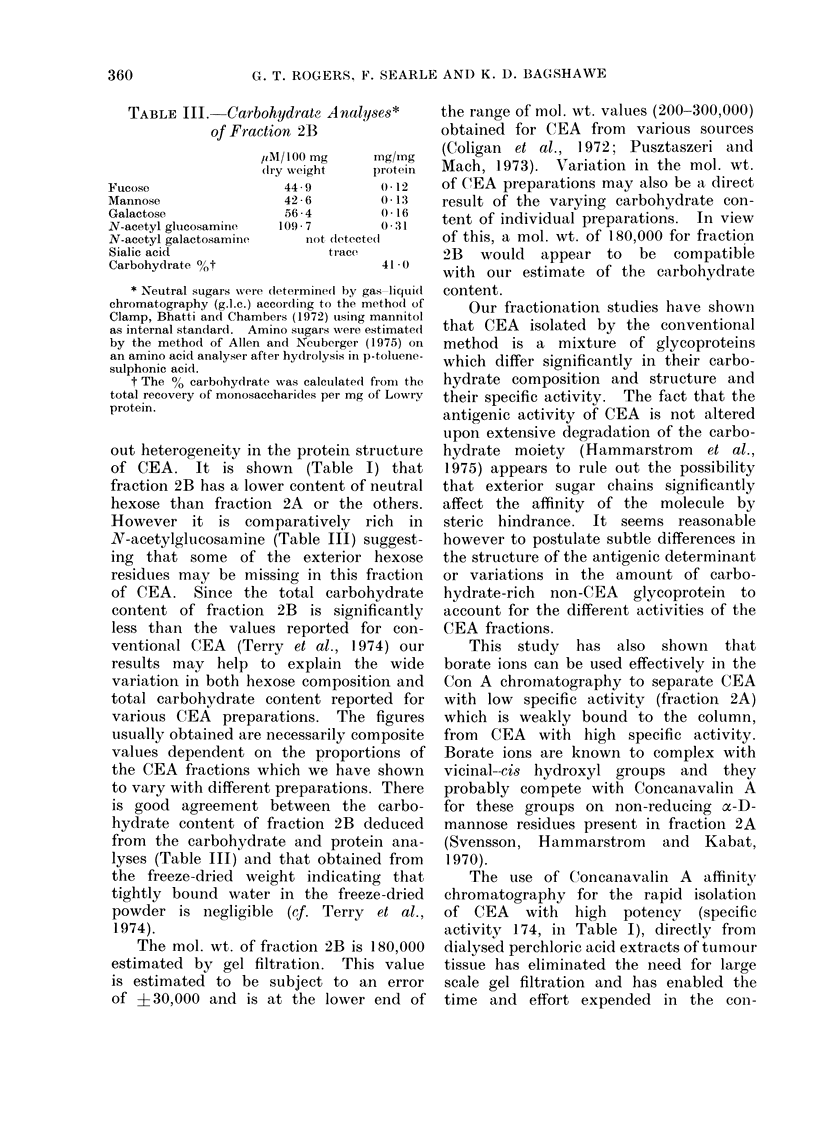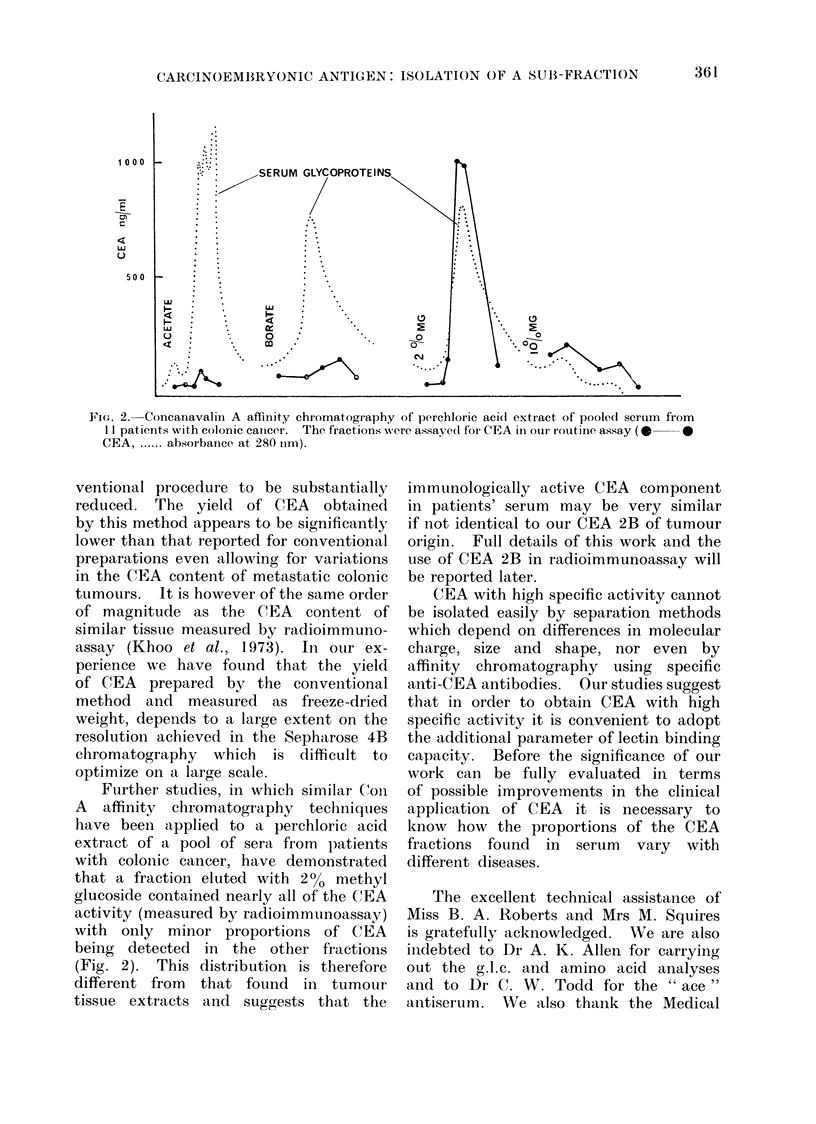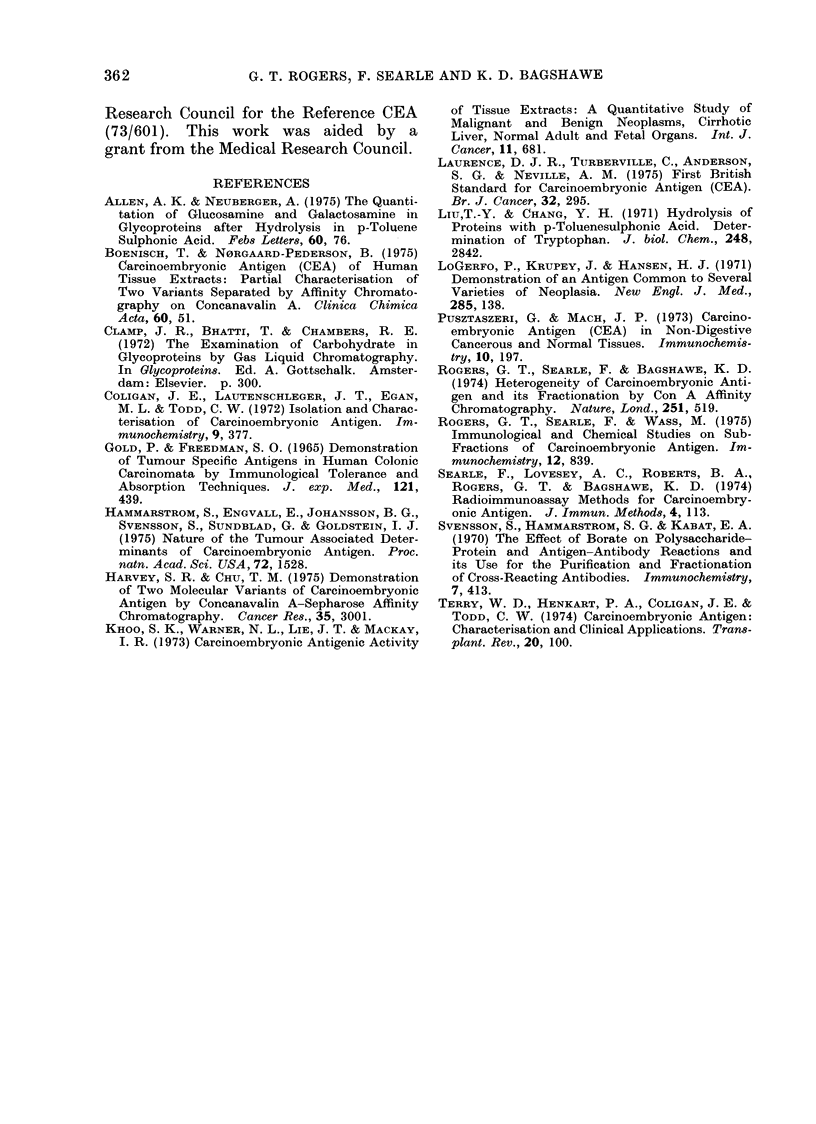# Carcinoembryonic antigen: isolation of a sub-fraction with high specific activity.

**DOI:** 10.1038/bjc.1976.59

**Published:** 1976-04

**Authors:** G. T. Rogers, F. Searle, K. D. Bagshawe

## Abstract

Four sub-fractions of carcinoembryonic antigen have been obtained by chromatography of conventional CEA on Con A-Sepharose and their immunoreactive contents have been determined. Comparative studies have shown that a fraction eluted with 2% methyl glucoside (CEA 2B) had the highest activity, with a potency (60 u/mug) twice that of unfractionated CEA although appreciable activity was also found in the other fractions. The amino acid composition of CEA 2B is similar to that reported for conventional CEA but there is a lower content of neutral hexoses and a comparatively high content of N-acetylglucosamine. Experiments with a pool of sera from 11 patients with colonic cancer, which had been fractionated on Con A-Sepharose, have shown that nearly all the CEA activity was contained in a fraction eluted with 2% methyl glucoside. A convenient method of isolating CEA with high specific activity directly from perchloric acid extracts of tumour tissue is also described.


*Br. J. Cancer* (1976) **33**, 357

### CARCINOEMBRYONIC ANTIGEN: ISOLATION OF A SUB-FRACTION WITH HIGH SPECIFIC ACTIVITY

G. T. ROGERS, F. SEARLE AND K. D. BAGSHAWE

*From the Department of Medical Oncology, Charing Cross Hospital, London W6 8RF*

Received 17 October 1975 Accepted 29 December 1975

**Summary.—Four sub-fractions of carcinoembryonic antigen have been obtained by chromatography of conventional CEA on Con A–Sepharose and their immunoreactive contents have been determined. Comparative studies have shown that a fraction eluted with 2% methyl glucoside (CEA 2B) had the highest activity, with a potency (60 u/μg) twice that of unfractionated CEA although appreciable activity was also found in the other fractions. The amino acid composition of CEA 2B is similar to that reported for conventional CEA but there is a lower content of neutral hexoses and a comparatively high content of *N*-acetylglucosamine. Experiments with a pool of sera from 11 patients with colonic cancer, which had been fractionated on Con A–Sepharose, have shown that nearly all the CEA activity was contained in a fraction eluted with 2% methyl glucoside. A convenient method of isolating CEA with high specific activity directly from perchloric acid extracts of tumour tissue is also described.**

CARCINOEMBRYONIC antigen (CEA), first isolated by Gold and Freedman (1965) is an immunoreactive macromolecular glycoprotein found on the surface of human colonic tumour cells. It can be detected in relatively high amounts in the sera of patients with metastatic colonic cancer although it is present in moderate to low amounts in the sera of patients with primary lesions of various cancers and with various non-malignant diseases (LoGerfo, Krupey and Hansen, 1971). The conventional method of isolating CEA involves perchloric acid extraction of colonic metastases followed by large scale exclusion chromatography on Sepharose 4B and Sephadex G-200 (Coligan *et al.*, 1972). Our recent studies (Rogers, Searle and Bagshawe, 1974; Rogers, Searle and Wass, 1975) have shown that CEA isolated in this way, and also its desialylated product, can be fractionated into 3 immunologically active sub-fractions using Concanavalin A affinity chromatography. Independent studies, which confirm some of our earlier results have since been reported (Boenisch and Nørgaard-Pederson, 1975; Harvey and Chu, 1975). Here we report on the further fractionation of CEA on Con A-Sepharose using sodium borate buffer and on the immunoreactive content of these fractions. The use of Con A affinity chromatography as a simple and effective way of isolating CEA with high specific activity is also described.

### MATERIALS AND METHODS

Unfractionated CEA was prepared from several metastatic colonic tumours using the method of Coligan *et al.*, 1972. The yield of our assay standard (M-12) prepared by this method was 30 mg protein/kg wet tissue. Unfractionated CEA was separated into 3 fractions on a column of Concanavalin A–Sepharose (Pharmacia) as previously described (Rogers *et al.*, 1974). Briefly, this involved elution of fraction 1 with 0·1 M sodium acetate buffer pH 6 containing 1 M NaCl, $10^{-3}$ M $CaCl_2$, $10^{-3}$ M $MgCl_2$ and

$10^{-3}$ M $MnCl_2$. Fractions 2 and 3 were then respectively eluted with 2% and 10% methyl glucoside solutions in the above buffer.

*Further fractionation of fraction 2.*—CEA fraction 2 (5 mg, measured as protein) after dialysis against the acetate buffer was concentrated to 2 ml using an Amicon PM 10 membrane and re-chromatographed on a fresh column of Con A–Sepharose which was eluted with 0·1 M sodium borate/phosphate buffer pH 6 to obtain fraction 2A and subsequently with 2% methyl glucoside solution in the acetate buffer to obtain fraction 2B. Starting with unfractionated CEA it was possible to obtain four fractions (1, 2A, 2B and 3) by successively eluting the column with the acetate buffer, the borate buffer, 2% methyl glucoside and 10% methyl glucoside.

*Radioimmunoassay of CEA.*—CEA activity of the fractions after dialysis against distilled water was determined by the double antibody assay using preparation M-12 as both standard and label, and "ace" 36 antiserum (Searle *et al.*, 1974). The relative potencies of unfractionated CEA, fraction 2B and the MRC reference preparation 73/601 were determined by calculating the percentage of inhibition of $^{125}$I-CEA in the presence of the unlabelled CEA. Glycoprotein concentration was expressed as freeze-dried weight obtained after drying over $P_2O_5$ except in the case of the MRC preparation where 10 $\mu$g of freeze-dried CEA powder is contained in 0·5 ml of reconstituted solution.

*Large scale isolation of CEA using Con A affinity chromatography.*—Liver metastases of colonic tumours (500 g) were homogenized in water and extracted with perchloric acid as previously described (Coligan *et al.*, 1972). The extract, after extensive dialysis against tap water, was concentrated using an Amicon PM 10 membrane (10 ml for each 500 g of tumour). An equal volume of the acetate buffer was then added and the solution passed through a 0·22 $\mu$m Millipore filter. The filtrate was applied to a column of Con A–Sepharose (96 ml) and eluted sequentially with the acetate buffer, the borate buffer, 2% methyl glucoside and 10% methyl glucoside. The fractions were monitored for CEA activity by Ouchterlony diffusion reactions using monospecific anti-CEA antiserum raised to unfractionated CEA, and by inhibition of $^{125}$I-labelled standard CEA–antibody reactions as employed in our routine double-antibody assay. High activity CEA was eluted with 2% methyl glucoside and concentrated to 2 ml. It was further purified on a calibrated column of Biogel P-200 (200 ml) which was equilibriated with 0·1 M phosphate buffered saline pH 4·5. The CEA was eluted as the first of two peaks soon after the void volume indicating a mol. wt. of 180,000. The sample was freeze-dried and assayed for CEA activity (Table I). The total yield was 2·8 mg.

### RESULTS AND DISCUSSION

The recovery of CEA protein in each fraction from the Con A affinity chromatography varied with different preparations. However, typical recoveries for fractions 1, 2A, 2B and 3 were 10, 10, 40 and 20% respectively. CEA fractions 2A and 2B produced a line of identity on double diffusion with anti-CEA antiserum although the line given by fraction 2B was always somewhat sharper. On a weight to weight basis fraction 2B had the highest immunological activity by direct measurement in our routine CEA assay although the activities detected in the other fractions were appreciable (Table I). The activity of fraction 2B has been confirmed by comparative inhibition assays demonstrating the ability of fraction 2B and our assay standard

TABLE I.—*Activity and Carbohydrate Content of CEA Fractions*

| Fraction | Activity (%)* | Neutral hexose† | Carbohydrate (%)‡ |
|---|---|---|---|
| Unfractionated | 100 | — | 56 |
| 1 | 23 | 1·18 | 70 |
| 2 | 170 | 0·39 | 42 |
| 2A | 66 | 0·66 | 42 |
| 2B | 196 | 0·31 | 38 |
| 3 | 110 | 0·51 | 44 |
| Large scale isolation | 174 | — | 39 |

* CEA activity expressed as % activity on a weight to weight basis of assay standard CEA (M-12).

† Neutral sugar including fucose was estimated by the orcinol sulphuric acid method and expressed as mg/mg Lowry protein.

‡ Total carbohydrate deduced from the freeze-dried weight after subtracting Lowry protein.

(M-12), measured as freeze-dried weights, and the MRC reference CEA (where 10 μg of CEA powder is contained in 0·5 ml of reconstituted solution) to inhibit the binding of unfractionated $^{125}$I-CEA by anti-CEA antiserum (Todd "ace" 36). Parallel inhibition curves are shown (Fig. 1) for the three activities. The potencies of preparations M-12 and fraction 2B were 30 and 60 units of CEA/μg of freeze-dried powder respectively. These preparations are therefore approximately 3× and 6× the potency of the MRC reference preparation where approximately 1·0 μg of freeze-dried CEA contains 10 units of activity (Laurence *et al.*, 1975). The above results show that the potency of conventional CEA preparations may vary considerably and also that it can be improved 2× by Concanavalin A affinity chromatography using the appropriate buffer systems.

The amino acid content (Table II) of fraction 2B is very similar to that reported for conventional CEA preparations (Terry *et al.*, 1974) indicating that the protein may be very similar, but there is no conclusive evidence ruling

TABLE II.—*Amino Acid Composition of CEA 2B*

| | Mol amino acid/$10^5$ g protein |
|---|---|
| Asp | 144·6 |
| Thr | 79·5 |
| Ser | 90·4 |
| Glu | 113·1 |
| Pro | 82·3 |
| Gly | 56·9 |
| Ala | 60·7 |
| ½ Cys | trace |
| Val | 66·0 |
| Met | 4·7 |
| Ile | 37·5 |
| Leu | 84·7 |
| Tyr | 33·3 |
| Phe | 33·8 |
| His | 14·3 |
| Lys | 43·6 |
| Arg | 31·9 |

Amino acids were determined by the method of Liu and Chang (1971) on a sample hydrolysed for 24 h at 100°C in 3 M p-toluenesulphonic acid. (Uncorrected figures.)

FIG. 1.—Comparative inhibition curves of CEA, M-12 (▼), Fraction 2B (○) and the MRC reference CEA (●) demonstrating the inhibition of binding of $^{125}$I-CEA (M-12) to goat anti-CEA "ace" 36.

TABLE III.—*Carbohydrate Analyses* of Fraction* 2B

| | μM/100 mg dry weight | mg/mg protein |
|---|---|---|
| Fucose | 44·9 | 0·12 |
| Mannose | 42·6 | 0·13 |
| Galactose | 56·4 | 0·16 |
| *N*-acetyl glucosamine | 109·7 | 0·31 |
| *N*-acetyl galactosamine | not detected | |
| Sialic acid | trace | |
| Carbohydrate %† | | 41·0 |

* Neutral sugars were determined by gas–liquid chromatography (g.l.c.) according to the method of Clamp, Bhatti and Chambers (1972) using mannitol as internal standard. Amino sugars were estimated by the method of Allen and Neuberger (1975) on an amino acid analyser after hydrolysis in p-toluene-sulphonic acid.

† The % carbohydrate was calculated from the total recovery of monosaccharides per mg of Lowry protein.

out heterogeneity in the protein structure of CEA. It is shown (Table I) that fraction 2B has a lower content of neutral hexose than fraction 2A or the others. However it is comparatively rich in *N*-acetylglucosamine (Table III) suggesting that some of the exterior hexose residues may be missing in this fraction of CEA. Since the total carbohydrate content of fraction 2B is significantly less than the values reported for conventional CEA (Terry *et al.*, 1974) our results may help to explain the wide variation in both hexose composition and total carbohydrate content reported for various CEA preparations. The figures usually obtained are necessarily composite values dependent on the proportions of the CEA fractions which we have shown to vary with different preparations. There is good agreement between the carbohydrate content of fraction 2B deduced from the carbohydrate and protein analyses (Table III) and that obtained from the freeze-dried weight indicating that tightly bound water in the freeze-dried powder is negligible (*cf.* Terry *et al.*, 1974).

The mol. wt. of fraction 2B is 180,000 estimated by gel filtration. This value is estimated to be subject to an error of ±30,000 and is at the lower end of the range of mol. wt. values (200–300,000) obtained for CEA from various sources (Coligan *et al.*, 1972; Pusztaszeri and Mach, 1973). Variation in the mol. wt. of CEA preparations may also be a direct result of the varying carbohydrate content of individual preparations. In view of this, a mol. wt. of 180,000 for fraction 2B would appear to be compatible with our estimate of the carbohydrate content.

Our fractionation studies have shown that CEA isolated by the conventional method is a mixture of glycoproteins which differ significantly in their carbohydrate composition and structure and their specific activity. The fact that the antigenic activity of CEA is not altered upon extensive degradation of the carbohydrate moiety (Hammarstrom *et al.*, 1975) appears to rule out the possibility that exterior sugar chains significantly affect the affinity of the molecule by steric hindrance. It seems reasonable however to postulate subtle differences in the structure of the antigenic determinant or variations in the amount of carbohydrate-rich non-CEA glycoprotein to account for the different activities of the CEA fractions.

This study has also shown that borate ions can be used effectively in the Con A chromatography to separate CEA with low specific activity (fraction 2A) which is weakly bound to the column, from CEA with high specific activity. Borate ions are known to complex with vicinal-*cis* hydroxyl groups and they probably compete with Concanavalin A for these groups on non-reducing α-D-mannose residues present in fraction 2A (Svensson, Hammarstrom and Kabat, 1970).

The use of Concanavalin A affinity chromatography for the rapid isolation of CEA with high potency (specific activity 174, in Table I), directly from dialysed perchloric acid extracts of tumour tissue has eliminated the need for large scale gel filtration and has enabled the time and effort expended in the con-

FIG. 2.—Concanavalin A affinity chromatography of perchloric acid extract of pooled serum from 11 patients with colonic cancer. The fractions were assayed for CEA in our routine assay (●——● CEA, ...... absorbance at 280 nm).

ventional procedure to be substantially reduced. The yield of CEA obtained by this method appears to be significantly lower than that reported for conventional preparations even allowing for variations in the CEA content of metastatic colonic tumours. It is however of the same order of magnitude as the CEA content of similar tissue measured by radioimmunoassay (Khoo *et al.*, 1973). In our experience we have found that the yield of CEA prepared by the conventional method and measured as freeze-dried weight, depends to a large extent on the resolution achieved in the Sepharose 4B chromatography which is difficult to optimize on a large scale.

Further studies, in which similar Con A affinity chromatography techniques have been applied to a perchloric acid extract of a pool of sera from patients with colonic cancer, have demonstrated that a fraction eluted with 2% methyl glucoside contained nearly all of the CEA activity (measured by radioimmunoassay) with only minor proportions of CEA being detected in the other fractions (Fig. 2). This distribution is therefore different from that found in tumour tissue extracts and suggests that the immunologically active CEA component in patients' serum may be very similar if not identical to our CEA 2B of tumour origin. Full details of this work and the use of CEA 2B in radioimmunoassay will be reported later.

CEA with high specific activity cannot be isolated easily by separation methods which depend on differences in molecular charge, size and shape, nor even by affinity chromatography using specific anti-CEA antibodies. Our studies suggest that in order to obtain CEA with high specific activity it is convenient to adopt the additional parameter of lectin binding capacity. Before the significance of our work can be fully evaluated in terms of possible improvements in the clinical application of CEA it is necessary to know how the proportions of the CEA fractions found in serum vary with different diseases.

The excellent technical assistance of Miss B. A. Roberts and Mrs M. Squires is gratefully acknowledged. We are also indebted to Dr A. K. Allen for carrying out the g.l.c. and amino acid analyses and to Dr C. W. Todd for the "ace" antiserum. We also thank the Medical

Research Council for the Reference CEA (73/601). This work was aided by a grant from the Medical Research Council.

REFERENCES

ALLEN, A. K. & NEUBERGER, A. (1975) The Quantitation of Glucosamine and Galactosamine in Glycoproteins after Hydrolysis in p-Toluene Sulphonic Acid. *Febs Letters*, **60**, 76.

BOENISCH, T. & NØRGAARD-PEDERSON, B. (1975) Carcinoembryonic Antigen (CEA) of Human Tissue Extracts: Partial Characterisation of Two Variants Separated by Affinity Chromatography on Concanavalin A. *Clinica Chimica Acta*, **60**, 51.

CLAMP, J. R., BHATTI, T. & CHAMBERS, R. E. (1972) The Examination of Carbohydrate in Glycoproteins by Gas Liquid Chromatography. In *Glycoproteins*. Ed. A. Gottschalk. Amsterdam: Elsevier. p. 300.

COLIGAN, J. E., LAUTENSCHLEGER, J. T., EGAN, M. L. & TODD, C. W. (1972) Isolation and Characterisation of Carcinoembryonic Antigen. *Immunochemistry*, **9**, 377.

GOLD, P. & FREEDMAN, S. O. (1965) Demonstration of Tumour Specific Antigens in Human Colonic Carcinomata by Immunological Tolerance and Absorption Techniques. *J. exp. Med.*, **121**, 439.

HAMMARSTROM, S., ENGVALL, E., JOHANSSON, B. G., SVENSSON, S., SUNDBLAD, G. & GOLDSTEIN, I. J. (1975) Nature of the Tumour Associated Determinants of Carcinoembryonic Antigen. *Proc. natn. Acad. Sci. USA*, **72**, 1528.

HARVEY, S. R. & CHU, T. M. (1975) Demonstration of Two Molecular Variants of Carcinoembryonic Antigen by Concanavalin A–Sepharose Affinity Chromatography. *Cancer Res.*, **35**, 3001.

KHOO, S. K., WARNER, N. L., LIE, J. T. & MACKAY, I. R. (1973) Carcinoembryonic Antigenic Activity of Tissue Extracts: A Quantitative Study of Malignant and Benign Neoplasms, Cirrhotic Liver, Normal Adult and Fetal Organs. *Int. J. Cancer*, **11**, 681.

LAURENCE, D. J. R., TURBERVILLE, C., ANDERSON, S. G. & NEVILLE, A. M. (1975) First British Standard for Carcinoembryonic Antigen (CEA). *Br. J. Cancer*, **32**, 295.

LIU, T.-Y. & CHANG, Y. H. (1971) Hydrolysis of Proteins with p-Toluenesulphonic Acid. Determination of Tryptophan. *J. biol. Chem.*, **248**, 2842.

LOGERFO, P., KRUPEY, J. & HANSEN, H. J. (1971) Demonstration of an Antigen Common to Several Varieties of Neoplasia. *New Engl. J. Med.*, **285**, 138.

PUSZTASZERI, G. & MACH, J. P. (1973) Carcinoembryonic Antigen (CEA) in Non-Digestive Cancerous and Normal Tissues. *Immunochemistry*, **10**, 197.

ROGERS, G. T., SEARLE, F. & BAGSHAWE, K. D. (1974) Heterogeneity of Carcinoembryonic Antigen and its Fractionation by Con A Affinity Chromatography. *Nature, Lond.*, **251**, 519.

ROGERS, G. T., SEARLE, F. & WASS, M. (1975) Immunological and Chemical Studies on Sub-Fractions of Carcinoembryonic Antigen. *Immunochemistry*, **12**, 839.

SEARLE, F., LOVESEY, A. C., ROBERTS, B. A., ROGERS, G. T. & BAGSHAWE, K. D. (1974) Radioimmunoassay Methods for Carcinoembryonic Antigen. *J. Immun. Methods*, **4**, 113.

SVENSSON, S., HAMMARSTROM, S. G. & KABAT, E. A. (1970) The Effect of Borate on Polysaccharide–Protein and Antigen–Antibody Reactions and its Use for the Purification and Fractionation of Cross-Reacting Antibodies. *Immunochemistry*, **7**, 413.

TERRY, W. D., HENKART, P. A., COLIGAN, J. E. & TODD, C. W. (1974) Carcinoembryonic Antigen: Characterisation and Clinical Applications. *Transplant. Rev.*, **20**, 100.